# On universal common ancestry, sequence similarity, and phylogenetic structure: the sins of P-values and the virtues of Bayesian evidence

**DOI:** 10.1186/1745-6150-6-60

**Published:** 2011-11-24

**Authors:** Douglas L Theobald

**Affiliations:** 1 Biochemistry Department, Brandeis University, Waltham, MA 02454, USA

## Abstract

**Background:**

The universal common ancestry (UCA) of all known life is a fundamental component of modern evolutionary theory, supported by a wide range of qualitative molecular evidence. Nevertheless, recently both the status and nature of UCA has been questioned. In earlier work I presented a formal, quantitative test of UCA in which model selection criteria overwhelmingly choose common ancestry over independent ancestry, based on a dataset of universally conserved proteins. These model-based tests are founded in likelihoodist and Bayesian probability theory, in opposition to classical frequentist null hypothesis tests such as Karlin-Altschul E-values for sequence similarity. In a recent comment, Koonin and Wolf (K&W) claim that the model preference for UCA is "a trivial consequence of significant sequence similarity". They support this claim with a computational simulation, derived from universally conserved proteins, which produces similar sequences lacking phylogenetic structure. The model selection tests prefer common ancestry for this artificial data set.

**Results:**

For the real universal protein sequences, hierarchical phylogenetic structure (induced by genealogical history) is the overriding reason for why the tests choose UCA; sequence similarity is a relatively minor factor. First, for cases of conflicting phylogenetic structure, the tests choose independent ancestry even with highly similar sequences. Second, certain models, like star trees and K&W's profile model (corresponding to their simulation), readily explain sequence similarity yet lack phylogenetic structure. However, these are extremely poor models for the real proteins, even worse than independent ancestry models, though they explain K&W's artificial data well. Finally, K&W's simulation is an implementation of a well-known phylogenetic model, and it produces sequences that mimic homologous proteins. Therefore the model selection tests work appropriately with the artificial data.

**Conclusions:**

For K&W's artificial protein data, sequence similarity is the predominant factor influencing the preference for common ancestry. In contrast, for the real proteins, model selection tests show that phylogenetic structure is much more important than sequence similarity. Hence, the model selection tests demonstrate that real universally conserved proteins are homologous, a conclusion based primarily on the specific nested patterns of correlations induced in genetically related protein sequences.

**Reviewers:**

This article was reviewed by Rob Knight, Robert Beiko (nominated by Peter Gogarten), and Michael Gilchrist.

## Background

In a recent study, I applied model selection theory to a data set of universally conserved protein sequences, in an attempt to formally quantify the phylogenetic evidence for and against the theory of universal common ancestry (UCA) [1]. For the conserved protein data, this study demonstrated that UCA is a much more probable model than competing independent ancestry models. One of the notable strengths of this study is that it provides evidence for common ancestry without recourse to the common assumption that a high degree of sequence similarity necessarily implies homology.

This UCA study was subsequently criticized in a paper by Koonin and Wolf (hereafter referred to as K&W), in which they argue that the results in favour of UCA are "a trivial consequence of significant sequence similarity between the analyzed proteins" and that my tests "yield results 'in support of common ancestry' for any sufficiently similar sequences" [2]. Here I show that K&W's conclusions are incorrect. While sequence similarity is a highly probable consequence of common ancestry, similarity alone is insufficient to establish homology by the model selection tests. Rather, the phylogenetic pattern of nested, hierarchical, sequence correlations is the dominant factor that forces the conclusion of common ancestry for the real protein data. Before considering K&W's specific arguments in detail, I give an extended background to the question of universal common ancestry and provide a setting for understanding why null hypothesis tests of significance, such as BLAST-style E-values, are inadequate to quantitatively address the evidence for and against UCA.

### Universal common ancestry: The qualitative evidence and need for a formal test

Universal common ancestry is the hypothesis that all extant terrestrial life shares a common genetic heritage. The classic arguments for common ancestry include many independent, converging lines of evidence from various fields, including biogeography, palaeontology, comparative morphology, developmental biology, and molecular biology [1,3-14]. The great majority of this evidence, however, is qualitative in nature and only directly addresses the relationships of limited sets of higher taxa, such as the common ancestry of metazoans or the common ancestry of plants.

The broader question of universal common ancestry is much more ambitious and correspondingly difficult to assess. Are Europeans, Euryarchaeota, Euglena, Yersinia, yew, and yeast all genetically related? Of course, biologists routinely incorporate all of these taxa into a universal phylogenetic tree, which is an explicit representation of the genealogical relationships among these diverse taxa. But any group of taxa can be connected in a tree; one can even make a phylogenetic tree from random sequences or characters. Yet is a tree itself justifiable in light of the evidence? In a paper that motivated my original test of common ancestry, Sober and Steel set out the issue very clearly [11]:

When biologists attempt to reconstruct the phylogenetic relationships that link a set of species, they usually *assume *that the taxa under study are genealogically related. Whether one uses cladistic parsimony, distance measures, or maximum likelihood methods, the typical question is *which *tree is the best one, not *whether *there is a tree in the first place.

This is the question I set out to answer: Is there a universal tree — or, more broadly, a universal pattern of genetic relatedness — in the first place?

Several researchers have recently questioned the nature and status of the theory of UCA or have emphasized the difficulties in testing a theory of such broad scope [11,15-18]. For example, Ford Doolittle has disputed whether objective evidence for UCA, as described by a universal tree, is possible even in principle:

Indeed, one is hard pressed to find some theory-free body of evidence that such a single universal pattern relating all life forms exists independently of our habit of thinking that it should [19].

This sentiment was echoed also by K&W, who concluded that a "formal demonstration of UCA … remains elusive and might not be feasible in principle." [2]. Such criticisms of UCA point to a need for a formal test, similar to the formal tests of fundamental physical theories like general relativity and quantum mechanics.

Darwin originally proposed UCA in 1859, yet was characteristically circumspect, only committing to the view that "animals are descended from at most only four or five progenitors, and plants from an equal or lesser number" [3]. The hypothesis of UCA was evidently an open question at least until the mid 1960's, when a debate about UCA and the universality of the genetic code (then as yet undeciphered) played out in the pages of Science. One of the most celebrated arguments for UCA is based on the fact that the genetic code is identical, or nearly so, in all known life. The argument had been circling informally for some years before Hinegardner and Engelberg first presented it in detail [20-23]:

Because the genetic code should remain invariant, its constancy can be used to establish the number of primordial ancestors from which all (present) organisms are derived. If, for example, the code is universal … then all existing organisms would be descendants of a single organism or species. If the code is not universal, the number of different codes should represent the number of different primordial ancestors …

Hinegardner and Engelberg's reasoning hinges on the assumption that the genetic code is so important for fundamental genetic processes that any mutations in the code would be lethal. Carl Woese criticized this argument, noting its dependence on the assumption that the genetic code is a "historical accident" and must not be "chemically determined" [23]. Woese was a proponent of the "stereochemical hypothesis", which holds that the association between a certain codon and its respective amino acid is dictated by chemical phenomena — that is, the observed code is required somehow by the laws of physics, perhaps by binding affinity of the nucleic acid codon to its corresponding amino acid [23-26]. Woese was also sceptical that the code was "frozen", and he postulated plausible mechanisms by which a degenerate code could evolve. If the code were somehow determined by physicochemical principles and evolvable, then multiple origins of life could conceivably converge independently on the same code. However, the stereochemical hypothesis was considered and largely disregarded by most researchers, including Francis Crick, due to a lack of evidence and difficulty in imagining a possible mechanism [20-22,24].

In 1968, Crick still presented the "frozen accident" argument for UCA with some reservation [24]. But by 1973, in his famous essay on the explanatory power of evolutionary theory, Theodosius Dobzhansky laid out the existing evidence for UCA as if it were beyond dispute [4]. According to Dobzhansky, the primary support for UCA is given by several key molecular similarities shared by all known life: (1) the "universal" genetic code, (2) nucleic acid as the genetic material, (3) shared polymers such as proteins, RNAs, lipids, and carbohydrates, and (4) core metabolism. These are today still the main arguments for UCA.

The standard presentation of this evidence is, however, strictly qualitative; it does not quantitatively assess the likelihood that these commonalities could be arrived at independently from multiple origins. Each of Dobzhansky's arguments for UCA has its weaknesses, and Sober and Steel provide several criticisms of these standard arguments [11]. While a detailed analysis of these lines of evidence for UCA is beyond the scope of this article, as a case study let us briefly revisit the "universal" genetic code, widely considered the most persuasive evidence for UCA [5,6,10,24].

The origin and evolution of the genetic code is still an open question [25,27], yet recently a modification of the stereochemical theory has made a comeback, in the form of the "escaped triplet hypothesis" of Yarus and Knight [28,29]. Their hypothesis has the great advantage that it is based on a large body of experimental data that has shown a significant chemical association between amino acids and their corresponding codon/anti-codon triplets found in RNA aptamers.

From Yarus and Knight's work, we now have empirical support for an association of specific codons with their respective amino acids, which means we have a viable mechanism for evolving similar or identical genetic codes from independent origins. Hence, the plausibility of a stereochemical theory decreases the force of the "frozen accident" argument for UCA — but by how much is unclear. Furthermore, we also know now that the genetic code is far from "frozen", and that it continues to evolve [27].

Ideally, we would like to evaluate the evidence for UCA from the universal genetic code, accounting for the frozen accident and stereochemical hypotheses and allowing for code evolution. For instance, we could calculate the following two different probabilities, and compare them: (1) the probability of arriving at a near universal genetic code assuming two or more independent origins and convergence due to chemical constraints from the escaped triplet hypothesis, versus (2) the probability of arriving at a near universal code assuming a single origin and a "frozen accident". Of course, reasonable and persuasive verbal arguments can be made on this point [25], and the qualitative evidence compellingly supports universal ancestry. But quantitatively estimating these probabilities is non-trivial. To calculate these probabilities we need formally specified stochastic models for how genetic codes evolve under both hypotheses — namely, we need well-defined likelihood functions, probability distributions for the observed data given each of the competing hypotheses. We currently have no such formal models for genetic code evolution [11,12,25,27]. The calculations are further complicated by the fact that there are other plausible hypotheses for the evolution of the code with empirical support [25,30], not all of which are mutually exclusive. Consequently, it is currently impractical to construct a formal, quantitative test of UCA using evidence from the genetic code.

Fortunately, however, we do in fact have ready-to-use, well defined, and widely accepted stochastic models for the evolution of protein sequences. These phylogenetic models of sequence evolution have been developed in detail over the past few decades in the field of molecular evolution, and they benefit from both a firm theoretical basis and widespread empirical support from genetics [31]. It is for these very reasons that I based my formal probabilistic, model-based tests of UCA on protein sequence data.

### Sequence similarity and homology are not equivalent

One common thread among the various arguments for common ancestry is the inference from certain biological similarities to homology. However, with apologies to Fisher, similarity is not homology. It is widely assumed that strong sequence similarity indicates genetic kinship. Nonetheless, as I and many others have argued [1,32-40], sequence similarity is strictly an empirical observation; homology, on the other hand, is a hypothesis intended to explain the similarity. Common ancestry is only one possible mechanism that results in similarity between sequences. In a landmark paper on the inference of homology from sequence similarity [33], the late Walter Fitch presented the problem as follows:

Now two proteins may appear similar because they descend with *di*vergence from a common ancestral gene (i.e., are homologous in a time-honoured meaning dating back at the least to Darwin's Origin of Species) or because they descend with *con*vergence from separate ancestral genes (i.e., are analogous). It is nevertheless possible that the restrictions imposed by a functional fitness may cause sufficient convergence to produce an apparent genetic relatedness. Therefore, the demonstration that two present-day sequences are significantly similar, by either chemical or genetic criteria, still must necessarily leave undecided the question whether their similarity is the result of a convergent process or all that remains from a divergent process. For example, it is at least philosophically possible to argue that fungal cytochromes c are not truly homologous to the metazoan cytochromes c, i.e., they just look homologous.

Colin Patterson made a similar argument [39], explicitly pointing out that statistically significant sequence similarity does not necessarily force the conclusion of homology:

… given that homologies are hypothetical, how do we test them? How do we decide that an observed similarity is a valid inference of common ancestry? If similarity must be discriminated from homology, its assessment (statistically significant or not, for example) is not necessarily synonymous with testing a hypothesis of homology.

How, then, would we know if highly similar biological sequences had independent origins or not? In all but the most trivial cases we do not have direct, independent evidence for homology — rather, we conventionally infer the answer based on some qualitative argument, often involving sequence similarity as a premise. One purpose of my original analysis [1] was to give this verbal argument a formal basis, using modern evolutionary models and probability theory. Perhaps the E-values from Karlin-Altschul statistics have already solved this problem from a statistical perspective [41-43]? As I explain in detail below, such is not the case. The most that a low E-value for sequence similarity (say, from a BLAST search) can do is show that the observed degree of sequence similarity is greater than that expected by random chance — the assessment of homology, on the other hand, is a distinct question. Furthermore, Karlin-Altschul tests for similarity miss a critical aspect of evolution that phylogenetic models readily grasp: the hierarchical structure of nested correlations induced in genetic sequences by genealogical processes.

### Logical problems and misconceptions with BLAST-style null hypothesis tests

A main motivation for my original analysis [1] was to escape from the logical quagmire posed by frequentist null hypothesis tests and bring state-of-the-art probabilistic methods (Bayesian and likelihoodist) to bear on the question of UCA. My tests of common ancestry are very pointedly conducted within a Bayesian and model-selection framework, eschewing frequentist null hypothesis methodology — a point made very prominently in the original report.

Nevertheless, in their criticism K&W state that they "interpret these tests [of common ancestry] within the null hypothesis framework." The reviewers for K&W's criticism similarly expect a null hypothesis in my analysis, ask whether I am using the Fisher or Neyman-Pearson methodology of null hypothesis testing, and claim that small P-values directly support a common ancestry hypothesis [2]. Due to these misconceptions about both my model selection methodology and the capabilities of frequentist null hypothesis tests, I find it necessary to explicitly lay out the advantages of the former and the logical problems with latter. Those who are familiar with Bayesian inference and model selection theory, and with the standard criticisms of frequentist null hypothesis tests, may wish to skip to the end of the *Introduction *where I specifically discuss K&W's simulation.

Frequentist hypothesis tests (of both Fisher and Neyman-Pearson flavors) have been highly criticized and disparaged for many decades by professional statisticians [44-71]. These criticisms are well-known in statistics — they in fact currently reflect the consensus view — and as such I make no claims of originality in the following. I begin with a representative quote from Jeff Gill, current Director of the Center for Applied Statistics at WUSTL, writing for social scientists, but the criticism applies generally:

The null hypothesis significance test (NHST) should not even exist, much less thrive as the dominant method for presenting statistical evidence in the social sciences. It is intellectually bankrupt and deeply flawed on logical and practical grounds. More than a few authors have convincing demonstrated this [54].

Gill goes on to list 33 high-profile supporting publications by mainstream statisticians from over the past half century.

#### E-values, P-values, and Karlin-Altschul statistics

A BLAST E-value is a Fisherian null hypothesis significance test [41-43,72]. With BLAST searches, the null hypothesis holds that the observed level of sequence similarity is an artefact generated by the optimal alignment of two random sequences [41-43,72].

To conduct a Fisherian null hypothesis significance test, a P-value is calculated based on a "null" distribution of some relevant statistic of the data. The P-value is interpreted as the weight of evidence against the null hypothesis; the smaller the P-value, the greater the evidence against the null. A P-value is defined as the probability of obtaining a value of the test statistic at least as extreme as the actual observed value, assuming that the null hypothesis is true. More formally, we say that the P-value = p(D|N), read as the conditional probability of D given (or assuming) N, where D is data equal to or more extreme than the observed value, and N is the null hypothesis.

**Figure 1:**
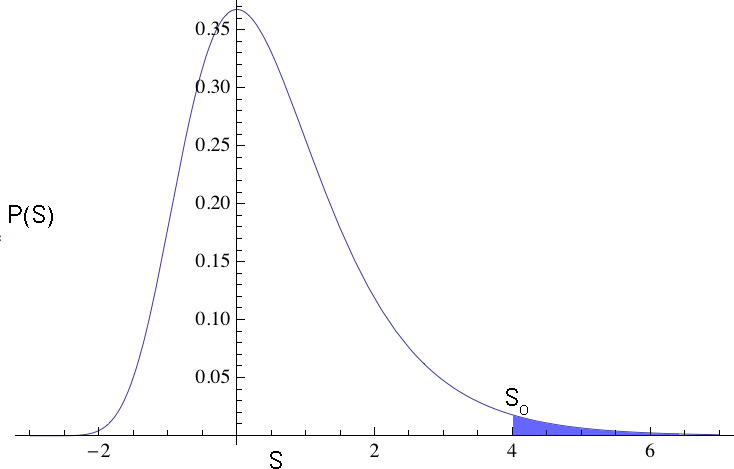
**Extreme value probability distribution for similarity scores**. A standard extreme value distribution (EVD) is shown. In Karlin-Altschul statistics, similarity scores (S) for alignments of random sequences are assumed to follow an EVD. P-values are based on the tail probability, shown as the shaded blue area, corresponding to the probability of observing a similarity score greater than or equal to the observed similarity score (S_o_) for a given alignment of interest.

In Karlin-Altschul statistics of sequence similarity, two sequences are optimally aligned by maximizing the similarity between them [41-43]. The similarity between two sequences is quantified by a similarity statistic, or similarity score, S. Conventionally, the similarity score is found by a weighted sum of the aligned positions between two sequences, in which the weights are given by a log-odds amino acid scoring matrix, such as the BLOSUM62 matrix [73]. If we take a large number of random sequences and align them (by maximizing the similarity score S), we will obtain a distribution of similarity scores. It has been found empirically that random similarity scores closely follow an extreme value distribution (EVD). An EVD has a bell-shaped curve, somewhat similar to a Gaussian, but is asymmetric with a longer right tail (see Figure 1).

For Karlin-Altschul statistics, then, the null distribution is the EVD of random similarity scores, where the similarity score is the appropriate test statistic. Imagine that we align two protein sequences and obtain the observed, optimal similarity score S for the alignment. The P-value is then the probability of that score or greater, as given by the EVD of random sequence alignments. This probability is also known as the "tail probability", as it quantifies the probability in the rightmost tail of the EVD (shown as the shaded region of the extreme value distribution in Figure 1).

Note that there is a close relationship between P-values and E-values. An E-value is similarly defined as the average number of times we expect to obtain a value of the test statistic at least as extreme as the value actually observed, assuming that the null hypothesis is true. For mostly historical reasons, the E-value is conventionally used more often in sequence similarity tests, like BLAST searches, and it can be directly calculated from the P-value:

(1) E= - ln(1 - P)

For small values of the P-value (< 0.05), both measures are approximately equivalent. The important point here is that for our purposes E- and P-values are interchangeable, and all the arguments made below apply equally to both.

As mentioned above, a low P-value (or E-value) is conventionally considered to be a measure of evidence against the null hypothesis; the smaller the P-value, the more reason we have to believe that the null is false. The logic behind this interpretation of the P-value is neatly summarized by Ronald Fisher's famous disjunction: Upon obtaining a small P-value, "*Either *an exceptionally rare chance has occurred, *or *the theory of random distribution is not true" (italics in original) [74]. For the moment, let us assume that this reasoning is valid — that a small P-value is indeed evidence against the null. We will revisit this assumption later.

Although the logical problems with null hypothesis statistical tests are many and profound, here I recount only three faults that are most pertinent to homology inference using a BLAST-style null hypothesis test for sequence similarity based on Karlin-Altschul statistics.

#### The False Dichotomy: Rejecting the null does not imply acceptance of a favoured alternative hypothesis

According to the logic of null hypothesis testing, a small P-value allows us to reject the null hypothesis at some specified "level of significance" [47,57,64,65,74-76]. By convention, a meaningful level of significance is often chosen as 0.05 or 0.01, though other values are frequently used depending on the application (e.g, PSI-BLAST by default uses a 0.005 cut-off [77]). P-values smaller than the chosen significance level allow us to "reject the null hypothesis" as false. Nevertheless, rejecting the null hypothesis of an alignment of random sequences is not logically equivalent to accepting common ancestry. This reasoning could be valid only if 'randomness' and 'common ancestry' were mutually exclusive hypotheses, but the logical complement of 'a random alignment' is 'not a random alignment', rather than 'common ancestry'. Non-random, significant sequence similarity can be due to many factors besides common ancestry.

The common belief that a small E-value indicates sequence homology (i.e., that the two sequences share a common ancestor) is based on a false dichotomy. During the 1981 creationist trial in Little Rock, Arkansas, the attorney for the State, David Williams, committed the same logical fallacy by arguing that criticisms of evolutionary theory were evidence for creationism. Geneticist Francisco J. Ayala, a witness for the plaintiffs, corrected him:

… negative criticisms of evolutionary theory, even if they carried some weight, are utterly irrelevant to the question of validity or legitimacy of creation science. Surely you realize that *not *being Mr Williams in no way entails *being *Mr Ayala! [78]

Clearly, I am not attorney Williams (with high statistical significance, P < 0.01), and just as clearly, the fact that we have established that I am not Williams does not imply that I am Dr Ayala. Likewise, *not *being an alignment of random sequences in no way entails *being *homologous.

If our aim is to establish my true identity, we could, one-by-one, rule out the possibilities that I am William Martin, or Eugene Koonin, or Yuri Wolf, or one of the other ~ 7 billion people on the planet. But wouldn't it be useful if we had a method that instead could directly provide positive evidence that I am Douglas Theobald? Unfortunately, null hypothesis significance tests are incapable by design of providing evidence for any hypothesis — null hypothesis tests are intended to only provide evidence *against *the null, no more nor less [75]. Hence null hypothesis tests, by their own frequentist logic, cannot provide evidence for common ancestry.

#### P-values cannot provide evidence for the null hypothesis

Furthermore, failing to reject the null, by obtaining a large P-value (e.g., P ≫ 0.05), does not imply that the null is true [75]. As Sir Ronald Fisher, inventor of the P-value and null hypothesis significance test, wrote: "It is a fallacy so well known as to be a *standard *example, to conclude from a test of significance that the null hypothesis is thereby established;… " (emphasis in original) [74]. Similarly, Fisher wrote that "it should be noted that the null hypothesis is never proved or established, but is possibly disproved, in the course of experimentation" so that "experimenters … are prepared to ignore all [insignificant] results" [79,80]. This is often stated as the maxim: "failing to reject the null does not mean accepting the null", or, more prosaically, "Thou shalt not draw inferences from a nonsignficant result!" [81]. For example, according to the logic of null hypothesis significance testing, an E-value of 10 does not mean that the sequence alignment is likely to be random — it simply means that a random alignment could easily have resulted in the observed level of sequence similarity. Clearly it would be beneficial to have a statistical method that could provide evidence *for *the null and not just evidence against it.

#### The "Prosecutor's Fallacy" and pregnant women: Improbable data does not imply an improbable hypothesis

Up till now, we have assumed that the frequentist position is correct, namely that Fisher's disjunction has logical force and a small P-value is evidence against the null hypothesis. But even this key premise is fallacious, as it relies on a notorious error of probabilistic reasoning known in law as the "Prosecutor's Fallacy" [82,83]. A small P-value, which measures the improbability of the data under the null, in fact does not imply that the null is improbable.

The Prosecutor's Fallacy arises from incorrectly inferring that a cause is unlikely because the effect is unlikely. A simple example may suffice to illuminate the problem. A quick glance around should establish that, at any given time, most women are not pregnant. In my state of Massachusetts, for example, the frequency of women that are pregnant is approximately 2% [84]. We can quantify this observation with a conditional probability statement: the probability that a particular person is pregnant, given that the person is a woman, is 0.02. Symbolically, we write p(P|W) = 0.02, where P is the proposition that "this person is pregnant" and W is the proposition that "this person is a woman". Note that p(P|W) is small — in fact small enough to be "statistically significant" by convention. Nevertheless, the small value for p(P|W) does not imply that the inverse probability, p(W|P), is also small. The probability that 'this person is a woman given that this person is pregnant' is obviously much greater than 0.02!

The same rules of probability necessarily apply to p(D|N) (the P-value) and the inverse probability p(N|D) (the probability of the null hypothesis given the observed data). Thus, a small P-value does not imply that the null hypothesis is unlikely. In fact, by itself, the P-value tells us nothing at all regarding the probability of the null hypothesis. The only way to calculate the probability of the null hypothesis, p(N|D), is by using Bayes theorem, but frequentist methodology does not allow us to do that.

The reason why the Prosecutor's Fallacy is false is simply because true hypotheses routinely predict low-probability data. Observing a piece of data that is unlikely could be nothing more than that — unlikely things happen all the time (like pregnant women or a winning lottery ticket). The null hypothesis may predict the observed data with a small probability, resulting in a small P-value, but if competing models are even worse — that is, competing models predict the same data with even *lower *probability — then why should we reject the null? For these reasons, there is no logical reason to think that the null hypothesis is likely to be false based solely on a small P-value; additional information is required.

### A way out: Bayes, likelihood, and model selection

Model selection methods, such as log likelihood ratios (LLR), the Akaike Information Criterion (AIC), and Bayes factors [47], are now used routinely in modern biological research, especially in phylogenetics, genetics, and bioinformatics [69,85-95]. Model selection theory has largely been developed as an alternative to null hypothesis significance tests, such as Fisherian P-values, which were developed within the frequentist statistics paradigm. The advantages of model selection methods include a firm logical and theoretical basis and elegant handling of model complexity, both of which make them attractive for analyzing complex evolutionary models for biological sequence data.

The core idea is elegantly simple: Quantitatively calculate how well different models explain the data (judged either by the likelihood of the data or by the posterior probability of a model), and compare the models to each other. Alternative models compete against each other head-to-head, and the observed data is the judge. From a likelihoodist point of view, such as when using the AIC, the preferred model is just the model that explains the data best (by assigning it the highest probability). From a Bayesian point of view, the preferred model is the model that has the highest probability given the data, within the set of models being evaluated. The critical part is in having explicit stochastic models (likelihood functions) for each of the hypotheses under comparison.

One great advantage of model selection methodology is how the complexity of competing hypotheses is handled. When judging the explanatory power of competing hypotheses, two opposing factors must be accounted for: parsimony and the fit to the observed data. By increasing the number of parameters in a model — i.e., by making the hypothesis more complex — one can always improve the "goodness of fit" to the data. For instance, when fitting a polynomial curve to a set of points, the discrepancy between the curve and the data points can be minimized arbitrarily by increasing the order of the polynomial until it equals the number of data points. However, simpler models are preferred, since increasing the complexity of the model increases the uncertainty in the estimates of the parameters. The fewer *ad hoc *parameters, the better — a principle informally known as Occam's Razor. P-values unfortunately have no way to account for model complexity. Model selection methods, in contrast, weigh these two opposing factors (goodness-of-fit and complexity) to find the hypothesis that is jointly the most accurate and the most precise.

Model selection methodology solves all the problems with null hypothesis significance test P-values enumerated above. First, in model selection methodology, there is no null hypothesis. All hypotheses are treated equally, none is given special favoured status, and they compete head-to-head. There is no need to rely on a false dichotomy, since multiple models are compared directly to each other. A hypothesis is "rejected" only if there is a better alternative. Second, model selection scores provide the evidence for each hypothesis, not just against the "null". The complexity and ability of a model to explain the data is the evidence for and against it, when compared to other competing hypotheses. Third, model selection methodology does not fall prey to the Prosecutor's fallacy. A particular model *A *may predict the data with a small probability, but if no other hypothesis does any better (after accounting for model complexity), then model *A *is the preferred hypothesis. Fortunately, in model selection theory pregnant women are not rejected.

In my formulation of the tests for UCA, independent ancestry models are represented by multiple phylogenetic trees, one tree for each group of taxa that is assumed to be genealogically related under this particular model [1]. Unrelated taxa are found in different trees. Because each tree is assumed to be independent (by definition of independent ancestry), the complete probability of the data assuming an independent ancestry model is simply the product of the probabilities of the several trees that compose it. For instance, consider two independent, unrelated trees of taxa *A *and *B*, and the sequence evidence *X*. The total probability of the sequence data *X *for the independent ancestry hypothesis *IA *is given by the joint probability of the data from tree *A *and tree *B*:

(2) p(X∣IA)=p(X∣A& B)=p(X∣A)p(X∣B)

Common ancestry models are represented by including all taxa in unified trees. Once 'common ancestry' and 'independent ancestry' are thus defined for a set of taxa, it is straightforward to apply the model selection tests to determine which hypothesis is best.

### Koonin and Wolf's rebuttal: Common ancestry models win in a simulation of similar sequences lacking phylogenetic structure

In a recent criticism of my model selection tests of UCA, Koonin and Wolf argue that the test results do not support the conclusion of universal common ancestry [1,2]. K&W support this claim by a simulation study in which similar sequences were randomly generated without any phylogenetic structure. In K&W's simulation, each column of a sequence alignment has a different, independent amino acid distribution (the amino acids are distributed according to a discrete distribution, also called a categorical distribution [96,97]). They then generated artificial sequences by randomly selecting amino acids from each column's distribution. The stochastic model corresponding to this simulation I will call the "profile" model, due to its similarity to common sequence profiles [98-100]. The profile model can be considered a star-tree in which each site has its own amino acid substitution matrix, and hence it cannot model phylogenetic structure or different levels of sequence similarity. K&W's simulated data has no phylogenetic signal in the sense that the sequences lack nested patterns of similarities. For this artificial data set generated by the profile model, the model selection tests choose common ancestry over independent ancestry.

K&W make three distinct claims based on their simulated data:

(1) the model selection tests prefer UCA solely due to the high similarity of the protein sequences, regardless of genealogical history,

(2) their simulation corresponds to a convergent, independent ancestry model, and therefore the model selection tests err in choosing common ancestry for the simulated sequences,

(3) the demonstration of UCA is dependent on the assumption that proteins with highly similar sequences share common ancestry.

All of these claims are incorrect, and I consider each of them in turn below.

## Results and Discussion

### Claim 1: The conclusion of common ancestry is primarily due to genealogical structure in the protein sequences, not mere similarity

In their Abstract, K&W make their main claim:

…the purported demonstration of the universal common ancestry is a trivial consequence of significant sequence similarity between the analyzed proteins. The nature and origin of this similarity are irrelevant for the prediction of "common ancestry" of by the model comparison approach.

Later they further explain that the model selection results in favour of UCA are "simply a restatement of the fact that these proteins display a highly statistically significant sequence similarity". Reviewer William Martin agrees: "They are absolutely right on this".

I present four different evidences demonstrating that K&W's primary claim — that my results are independent of phylogenetic history and are simply due to sequence similarity — is incorrect. The first piece of evidence is based on mathematical considerations of the theory behind the phylogenetic models used in the tests, while the final three are empirical.

#### I. Phylogenetic models involve more than mere sequence similarity

Based on the theory underlying modern phylogenetic models, we know that sequence similarity is only one component that indirectly affects the model selection tests [31,101,102]. Each of the models, whether common ancestry or independent ancestry, involve phylogenetic trees. Trees are mathematical structures that can account for both gross similarity (largely in the branch lengths) and subtle patterns of similarities (in the particular topology of the tree) [31,101,102]. It is this latter component — the complex hierarchical patterns of similarities induced by a genealogical branching process — that phylogenetic trees can account for but methods like BLAST, which consider only overall sequence similarity, cannot.

**Figure 2:**
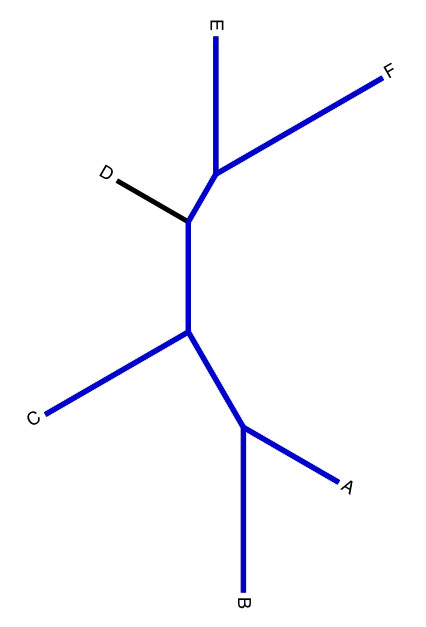
**Example phylogeny**. A toy phylogeny of six sequences, represented by the letters A-F.

Consider the phylogenetic tree of six protein sequences shown in Figure 2. In standard Markovian phylogenetic models, such as the ones used in the model selection tests, the probability of the six sequences depends on the topology of the tree and its branch lengths [31,101]. The sum of the branch lengths between two sequences is proportional to the number of evolutionary substitutions separating those two sequences. In this sense, the total distance along the tree between two sequences is a measure of their similarity. Importantly, if you change the topology, while maintaining the same distance between two sequences, or even maintaining the same total tree length, you generally change the likelihood of the model (i.e., you change the probability of the sequences).

Now imagine that you replace these six sequences with a different set that nevertheless has identical percent identity and similarity as the original set (perhaps as measured by an alignment score using a substitution matrix). In general this replacement will also change the likelihood, *even if the topology and branch lengths are held constant*. This means that the likelihood of a tree is not simply a function of the similarities of the sequences [31,101,102]. Rather, the likelihood of a phylogenetic tree is also a function of the nested pattern of similarities in the data. Therefore, the model selection tests, which are explicitly based on likelihoods of trees, consider information in the sequences that cannot be reduced to simple sequence similarity.

Hence it is possible for highly similar sequences to nevertheless have conflicting hierarchical structure that does not fit well to a single, global tree. Common ancestry implies phylogenetic structure; Markovian character evolution along a bifurcating tree results in hierarchical patterns of correlated character changes. If the phylogenetic structure in a set of highly similar sequences is conflicting, then this is evidence against common ancestry, and it could be evidence for independent ancestry models that do not force the proteins into a global phylogeny. This is one key advantage of my model-based tests over simple BLAST-type analyses that only look at gross sequence similarity.

K&W quote what they call a "key sentence" from my Nature letter where I explain the significance of the large test scores favouring UCA:

… when comparing a common-ancestry model to a multiple-ancestry model, the large test scores are a direct measure of the increase in our ability to accurately predict the sequence of a genealogically related protein relative to an unrelated protein.

Later they claim that this sentence is "simply a restatement of the fact that these proteins display a highly statistically significant sequence similarity". However, from the considerations given above, we know that a phylogenetic model's ability to predict a given sequence (e.g., sequence D in Figure 2) from other sequences (e.g. sequences A, B, C, E, F in Figure 2) is a function of the tree topology and the patterns in the sequences, not simply of the gross similarities among the sequences.

#### II. High sequence similarity is insufficient to force the conclusion of common ancestry

If K&W's hypothesis is correct — that the model selection tests choose common ancestry simply due to sequence similarity — then the tests should choose common ancestry over independent ancestry for any set of sequences with highly statistically significant sequence similarity. K&W make this very claim in their Discussion: "The likelihood tests of the kind described by Theobald … yield results 'in support of common ancestry' for any sufficiently similar sequences." However, this prediction is directly contradicted by the example given in the Supplementary material of my original Nature letter (section 4.3, pages 16-20 [1]). There I present a simple case in which the model selection tests choose independent ancestry for sequences with highly significant similarity. Here I present another similar example, in which four sequences have highly significant similarity to each other (in all possible pairwise comparisons, Table 1), and yet the model selection tests prefer independent ancestry models over common ancestry (Table 2). Therefore, the model selection tests necessarily consider factors other than mere sequence similarity, and highly significant sequence similarity is not sufficient for the model selection tests to choose common ancestry.

**Table 1:** Significant similarity among four artificially constructed sequences.

Sequence	E	M	P
B	4e-50	1e-17	1e-27

E		4e-31	1e-17

M			2e-36

E-values from pairwise BLAST comparisons between four protein sequences (called B, E, M, and P), as reported by the NCBI bl2seq utility (see Appendix 2, Additional File 1 for the sequences).

**Table 2:** Model selection scores for four artificially constructed, significantly similar sequences.

Model	ln Lik	LLR	K	ΔAIC
CA (BEMP)	-5411	0	25	0

IA (BE+MP)	-5092	-319	44	-300

IA (BM+EP)	-5009	-402	44	-383

IA (BP+EM)	-5038	-373	44	-354

IA (E+BMP)	-5115	-296	42	-279

IA (M+BEP)	-5128	-283	42	-266

IA (P+BEM)	-5060	-351	42	-334

IA (B+EMP)	-5022	-389	42	-372

Common ancestry is compared with various independent ancestry models for four sequences with highly significant similarity to each other (see Appendix 2, Additional File 1 for the sequences). CA, common ancestry; IA, independent ancestry; LLR, log-likelihood ratio; K, number of parameters in model; AIC, Akaike information criterion. Model selection scores are relative to the common ancestry model; negative scores (LLR and ΔAIC) indicate better models. Independent ancestry model "BE+MP" indicates that B and E are homologous to each other, and that M and P are homologous to each other, yet B and E have an independent ancestry from M and P.

Why do the model selection tests favour independent origins for these sets of sequences, in spite of significant similarity? The answer is that these similar sequences have conflicting phylogenetic structure, and conflicting phylogenetic correlations are unlikely to have been generated by a common ancestry process. The known presence of conflicting phylogenetic structure in the universal protein data set I used [103] (as indicated by suspected horizontal gene transfer events) was in fact a major motivation for my model selection tests of UCA — it is possible that a high enough degree of conflicting phylogenetic structure could indicate that a particular independent ancestry hypothesis is a superior model for the universal protein data.

#### III. Star tree models account for similarity, but not for genealogical structure in the real protein data

K&W suggest that the universal proteins in my dataset could have been generated by a process completely lacking all phylogenetic structure, namely their profile model. However, there are very compelling a *priori *reasons to suspect that K&W's hypothesis should be false. The strong phylogenetic structure in the universal protein data set I used (and in similar data sets in the literature) is well established, and trees based on this dataset have local regions of high topological support [103,104]. Nevertheless, it would be useful to quantify the amount and influence of phylogenetic structure, as opposed to mere sequence similarity, in the universal protein data.

One way to formally test for phylogenetic structure is to see how well the data is explained by a star tree. Star trees model varying degrees of similarity among a set of proteins, as each branch in the star tree can be a different length, but star trees cannot account for hierarchical genealogical patterns. In fact, according to the model selection criteria, star trees explain K&W's simulated data much better than independent ancestry models (by several hundred log likelihood units, see Table 3). In contrast, star-tree models are extremely poor models for the universal protein data — they are actually worse than independent ancestry models (differences in log-likelihood greater than 20,000, see Table 4). Thus, the universal proteins display significant genealogical structure that is even more important than sequence similarity.

**Table 3:** Model selection scores for Koonin and Wolf's artificial data.

Model	ln marginal lik (+/- SEM)	ln BF
profile	-7521 (21)	0

CA	-7646 (20)	125

Star	-7813 (20)	292

IA	-8164 (14)	643

Log marginal likelihoods and Bayes factors for Koonin and Wolf's artificial data. Values reported are averages and standard error of the mean (SEM) for the 100 simulated alignments. CA, common ancestry; IA, independent ancestry; BF, Bayes factor

**Table 4:** Model selection scores for the universal protein data.

Model	ln marginal lik	ln BF
CA (ABE)	-126,713	0

IA (AE+B)	-133,602	6,889

IA (AB+E)	-134,744	8,031

IA (BE+A)	-135,201	8,488

IA (ABE_M +M)	-138,899	12,186

IA (A+B+E)	-140,578	13,865

IA (ABE_H +H)	-140,713	14,001

star	-148,883	22,170

profile	-151,145	24,432

Log marginal likelihoods and Bayes factors for the real, universal protein data set.

#### IV. Koonin and Wolf's "sequence similarity only" profile model does not explain the universal protein data

Most importantly, we can directly test K&W's profile model against both common ancestry and independent ancestry models and see if the profile model can adequately explain the sequence data. The profile model can account for neither phylogenetic structure nor differing degrees of sequence similarity, as it is essentially a symmetrical star tree where each site has a different substitution matrix of amino acid exchangeabilities. The profile model is something of a pathological case, since for these datasets there are roughly as many parameters as data points (19 parameters per column, giving a data-to-parameter ratio of 1.05 for K&W's artificial dataset, 0.63 for the universal protein dataset). The low data-to-parameter ratio invalidates the AIC and non-nested LLR tests, as it strongly violates the approximations needed to justify the tests (the AIC and LLR are strictly only valid in the asymptotic limit of infinite data) [94,105]. Bayes factors, however, do not suffer from this limitation [106], and there is a conveniently simple analytic expression for the marginal likelihood of the profile model (see the Appendix, Additional File 1).

According to the Bayes factors, K&W's artificial data is best explained by the profile models, which are preferred over the common ancestry models (see Table 3). This is expected, as K&W's artificial data was simulated under the profile model. However, for the universal protein data set used in my paper, the profile models are extremely poor compared to common ancestry (log-likelihood differences 30,000, Table 4) — much worse even than independent ancestry models. Therefore, contrary to K&W's hypothesis, similarity alone cannot explain the complex sequence correlations observed in the universal protein data set.

#### For the real data, hypotheses that model phylogenetic structure are best, those that model sequence similarity alone are worst

For the real, universal data set, the models are ordered as follows, best to worst: common ancestry > independent ancestry > star tree > profile model. This ordering indicates that phylogenetic structure is an extremely important factor for the real data set, actually more important than gross sequence similarity (unlike K&W's artificial data set). The common ancestry models can adequately account for phylogenetic structure and sequence similarity, so they are best. The independent ancestry models also can account for the phylogenetic structure within each independent group of related sequences cannot account for similarity between the groups. Nevertheless, independent ancestry models are better than the star tree and profile models, which can account for sequence similarity but lack all phylogenetic structure.

For K&W's artificial data, in contrast, the profile model is best and the independent ancestry model is worst. K&W's simulated data has no phylogenetic, hierarchical structure. The profile model is the true generating model for the simulated data, and it accounts for the different evolutionary processes at each site in a way the other models cannot, so clearly it should be preferred. Since sequence similarity is the only factor in their artificial data set, the independent ancestry models will be the worst, as they cannot adequately account for sequence similarity between two or more groups. The fact that common ancestry models are preferred over independent ancestry is also unsurprising. As discussed below, K&W's profile model is a common ancestry model, and therefore produces artificial sequences that mimic homologous sequences.

### Claim 2: Koonin and Wolf's simulation corresponds to a common ancestry model

K&W assert, without proof, that the profile model used in their simulation produces "phylogenetically unrelated sequences". Hence they believe they have presented a counterexample where my model selection tests artifactually choose common ancestry for convergent proteins. Reviewer Ivan Iossifov (of the K&W comment) agrees and claims that K&W "perform a simulation experiment which clearly demonstrates… Theobald's method chooses UCA hypothesis with virtual certainty over data generated by a convergent evolution model." However, K&W never state the assumptions of their proposed convergent hypothesis, nor do they derive a stochastic model from those assumptions. We therefore have no reason to believe that the simulation generates artificial convergent proteins. Rather, as detailed below, the simulated sequences mimic homologous proteins.

#### Homologous sequences may be statistically independent

First, consider the justification K&W provide for why they their simulation produces convergent sequences:

… these [simulated] alignments contain no signal of common ancestry (in more general terms, no evolutionary signal) whatsoever because each position in each sequence is generated independently from other positions.

If this claim were true, it would invalidate nearly all of modern phylogenetics, since the standard evolutionary models assume site independence, i.e., that the evolutionary change at each position is independent of other positions [31,101]. The "independent sites" assumption is admittedly a convenient approximation, a simplification that is likely valid for a large majority of potential site interactions but invalid for a small fraction of interacting sites. However, there is no reason that different sites in homologous proteins cannot evolve independently (many do), and therefore the fact that each position in K&W's simulated alignments is independent in no way implies non-homology.

#### K&W's profile was constructed from likely homologs

To produce their simulated sequences, K&W constructed a frequency profile from a large set of universally conserved proteins that most biologists believe to be homologous (including Koonin, Wolf, and their reviewers [2]). At the very least, the fact that their simulation method is based on real universally conserved proteins suggests that sequences generated by their simulation will reflect, to some extent, whatever real process actually produced those conserved proteins. If, to take an obvious example, the proteins they based the simulation on are indeed homologous, then sequences produced by K&W's simulation method will be strongly biased to look like bona fide homologous sequences.

#### The simulated alignments were produced by a well-known common ancestry model

In fact, the K&W profile model is a special case of a common ancestry model that has been described previously in the phylogenetic literature. K&W's profile model is a phylogenetic, common ancestry model, where each site has its own reversible amino acid substitution process and independent set of equilibrium frequencies, in which the proteins have evolved according to a star tree with equal branch lengths. This model was first considered by Bruno in 1996 [98,107]. Lartillot and Philippe 2004 refer to it as the MAX-Poisson model with uniform site rates (it is a special case of their CAT models), and they implement it in the phylogenetic software PhyloBayes [100] and PhyML-CAT [108]. Hence, the K&W simulation is invalid as a counter-example, since my model selection tests correctly choose common ancestry over independent ancestry for the simulated homologs.

#### A common ancestry model based on homologous sequences should not simulate convergent proteins

With these points in mind, let me restate exactly what K&W did in their simulation. They constructed a frequency profile from a large set of universally conserved proteins that most biologists believe to be homologous. Then they used this profile to generate sequences with the MAX-Poisson common ancestry model. Finally, they performed my model selection tests on this artificial sequence data set, finding that the tests favour common ancestry. It is difficult to understand how one could possibly conclude from this simulation that the model selection tests are behaving improperly.

K&W's simulated sequences lack phylogenetic signal in the sense that the sequences lack correlated hierarchical structure. But lacking hierarchical structure does not necessarily imply that the sequences are independent evolutionary inventions (though a lack of phylogenetic structure may decrease the probability of common ancestry relative to competing hypotheses that predict a lack of structure). As mentioned earlier, star trees are bona fide common ancestry models that lack hierarchical structure. A star tree is a phylogeny, after all.

### Claim 3: The conclusion of homology does not involve circular logic

Several critics, including K&W and their reviewer Arcady Mushegian [2], have suggested that the model selection tests for common ancestry implicitly involve "hidden" circular logic of some sort. Here I consider several of the potential sources of circularity, including issues involving sequence similarity, models of sequence evolution, and sequence alignment.

#### Model selection tests do not assume that sequence similarity implies homology

K&W claim that the conclusion of UCA, based on my model selection tests, requires "the assumption that universally conserved orthologous proteins with highly similar sequences actually originate from common ancestral forms". However, this contradicts the logic of model selection tests. Common ancestry models of course assume that the sequences they model are homologous. On the other hand, independent ancestry models assume that only certain subsets of sequences are homologous. The only other assumptions made by these models are the standard phylogenetic assumptions: that the evolution of homologous proteins proceeds by a standard Markovian model of sequence evolution (that is time-stationary, reversible, and site-homogeneous), that each site in the proteins is independent, and that new homologous proteins are generated by gene duplication events (lineage splitting) represented by bifurcating trees. Importantly, however, there is no assumption about which of these models is correct, and there is no assumption that "highly similar sequences are homologous". The winning model is dictated, objectively, by how well it explains the data relative to the other models. In other words, the model selection tests tell us which model, and hence which set of corresponding assumptions underlying that model, is best. The results could have pointed to independent ancestry, but that's not the answer this real data set gave.

#### Using amino acid substitution matrices does not imply an assumption of homology

One of the main assumptions underlying the phylogenetic models is that homologous sequences have evolved by a Markovian process of amino acid residue substitution, described by a 20 × 20 matrix of substitution rates. The popular rate matrices, such as WAG [109] and LG [110], were originally constructed from large alignments of ostensibly homologous protein sequences. However, for the formal purposes of the model selection tests, we do not actually know that the rate matrices were based on homologous sequences. We do know that similar sequences were used in constructing the rate matrices, but recall that similarity is not necessarily homology. From a model-selection perspective, the origin of the substitution matrices is irrelevant — the likelihood function does not know where the matrices came from, and the likelihood is the final judge. All that matters is that some substitution matrices result in higher model selection scores than others. The origin of a hypothesis is of no relevance to how it fairs when tested in a model selection method (arguing otherwise would be committing a type of "genetic fallacy"). Furthermore, the majority of the variance in rates in a substitution matrix can be captured by a single chemical property, namely hydrophobicity [111]. This strongly suggests that the differing rates in a substitution matrix are due to biophysical factors, rather than common ancestry (as first discussed by Zuckerkandl and Pauling [14]).

#### A sequence alignment does not necessarily represent homologous sites

Sequence alignments are often interpreted as representing a homology hypothesis, since each column in an alignment can show which residues in a sequence are related by common ancestry. Under this interpretation, using a fixed alignment in the model selection tests for common ancestry may seem to assume common ancestry from the outset. But this is only one possible interpretation of a sequence alignment. The similarities shown in an alignment between specific residues may be due to homology or analogy. Sequence alignment is nothing more than a procedure for maximizing similarity, and it is unnecessary to assume common ancestry to maximize similarity.

As a concrete example, it is common practice in SELEX experiments to align RNA aptamer sequences (with themselves and with natural sequences) in order to find common motifs, in spite of the fact that the aptamer RNAs may be known to each have independent origins from a random sequence library [112]. In this case aligned positions do not represent homologous residues, but rather functionally analogous residues. Similarly, one can align promoter sequences to find common motifs, without assuming that the promoters actually originated by descent from a common ancestral sequence [113]. An alignment can also be generated from pure three-dimensional structural data, even for proteins known to be unrelated [114,115]. The resulting structure-based alignment depicts structurally analogous residues, not homology. Therefore, an alignment is not necessarily a proposal of homology, because similar aligned sequences may be merely structurally or functionally analogous.

#### Alignment algorithms can bias the tests towards common ancestry with weakly similar sequences

It is possible that the process of sequence alignment can bias the results of the tests towards common ancestry [116]. By maximizing residue matches, sequence alignment algorithms can artifactually induce similarities between unrelated sequences. In certain extreme cases these artifactual similarities can prejudice the model selection tests towards common ancestry if the alignment bias is large and unaccounted for.

The effect of alignment bias is maximal for random sequences where it results in a highly uncertain alignment with spurious similarities; it is minimal for sequences of high similarity where the alignment is relatively certain; and the bias vanishes in the limiting case of long identical sequences where no alignment uncertainty exists. Accounting for alignment bias and uncertainty would be an important consideration for testing homology hypotheses in cases of very little sequence similarity. However, it has a negligible effect on the tests of common ancestry reported in my original Nature letter [1], which were based on universally conserved proteins with relatively high sequence similarity. Furthermore, the Brown dataset used in my original report had ambiguously aligned regions removed [103], including all indels, essentially eliminating any potential alignment bias and resulting in a very high confidence alignment.

If alignment bias is suspected to be a significant factor for a set of dissimilar sequences, then there are several ways to account for it using the model selection tests for common ancestry.

First, in a Bayesian framework, one can simply substitute BAli-Phy [117] for MrBayes [118] in the model selection tests. BAli-Phy elegantly handles alignment bias and uncertainty by treating the alignment as a parameter and integrating over it. BAli-Phy is unfortunately much more computationally intensive than using MrBayes, and thus should be used only when absolutely necessary. Nevertheless, I have repeated the Class II Bayes factor model selection tests of UCA with BAli-Phy, and the results closely recapitulate those using MrBayes, as fully expected when using similar sequences.

Second, one can rather easily estimate an upper bound for the effect of alignment bias by randomly permuting the sequences, realigning them, and performing the model selection tests with PhyML [119] or MrBayes. The permuted and realigned sequences have no phylogenetic structure, and all similarities in the realignment are solely due to the alignment procedure. The preference for UCA with the randomly permuted sequences, as measured by the model selection scores, is then an estimate of the maximum amount that the alignment procedure artifactually biases the results towards homology. This control was performed for the universal protein data, and the bias was found to be negligible compared to the true (unpermuted) model selection scores (hundreds of log-likelihood units versus thousands, respectively, indicating a maximum ~10% error in the reported model selection scores).

Finally, if high-resolution structures are available, one can use structure-based alignments, which do not optimize sequence similarity and hence avoid sequence alignment bias altogether.

## Conclusions

The various results reported here provide a direct refutation of the claim that model selection preference for UCA is merely a consequence of high sequence similarity [2]. Unlike star trees and K&W's profile model, which can only explain sequence similarity, the common ancestry models can also explain nested hierarchical sequence correlations. Hierarchical structure in a set of sequences is a direct prediction of genealogical models, in which branching of lineages occurs. For the real, universal protein data set, this hierarchical structure is the overriding factor for the superiority of the UCA hypothesis over competing hypotheses, including various independent ancestry models.

Moreover, the models used in the tests do not assume a priori that significant sequence similarity implies homology. Rather, sequence similarity is a consequence of the common ancestry models, and the common ancestry models simply explain the data best. Hence, the strong results from the model selection tests provide a firm logical basis for relying on the inference from sequence similarity to homology as a general principle, as long as there is no strongly conflicting phylogenetic structure.

As in all of science, our best theories may be incorrect. It is always possible that a biological model may be proposed in the future that explains the data better than the UCA models. Clearly I have not tested all possible models, especially those yet to be developed. I emphasize again here, as I have elsewhere [120], that I have not provided absolute "proof" of UCA. Proof is for mathematics and whiskey; it is not found in science. Nevertheless, these results provide strong evidence for UCA, given the hypotheses and sequence data currently available. As it stands, UCA explains the data best by far. With the model selection framework it is easy to test a novel, properly specified hypothesis against the common ancestry models. The profile model proposed by Koonin and Wolf, however, fails the test.

## Methods

Model selection tests were performed as described [1,2]. Star tree models were analyzed using a modified MrBayes [121], in which a star tree was given to MrBayes as an initial tree and topology rearrangements were prohibited. Terminal branch lengths were sampled. Similar log-likelihoods were found, independently, by PROML from the PHYLIP phylogenetics package [122], which allows a star tree as a constrained topology in a maximum likelihood analysis (this method also found AIC scores similar to the Bayes factors reported in Tables 3 and 4). For the Koonin and Wolf profile model, marginal likelihoods were calculated using Equation 12 in Appendix 1 (see Additional File 1). Koonin and Wolf's simulated alignments [2] were generously provided by Eugene Koonin.

### Reviewers' comments

#### Reviewer 1: Rob Knight

In this manuscript, the author responds to criticisms of his prior work that provides a Bayesian framework for formally testing the hypothesis that all life descends from a single common ancestor. Specifically, Koonin and Wolf provided a set of simulations that demonstrate that the same test will incorrectly identify sequences with similar composition that are independently generated as being related. In this paper, several additional tests are provided, including the demonstration that star phylogenies (in which all sequences stem from a single common ancestor with no branching structure) explain the simulated data but not real sequence data, as does a profile model in which sequences are independently generated from a profile with no phylogenetic structure. The key conceptual advance is to perform model selection, rather than to test deviations from a null hypothesis that may be inappropriate.

Overall, the calculations are convincing, although the article could benefit from being rewritten in a less combative tone and as a less narrow response to a single article. I am also somewhat unconvinced by the relevance of the material on the genetic code to the problem of convergent versus divergent protein evolution: a better example might be the repeated evolution of the same Trp or Ile active site, each of which has been demonstrated many times in different selections, which could provide the basis for truly independent evolutionary origins and which might therefore be interesting to analyze in the context of the present framework. The so-called "Welch-mer" also provides a fascinating example of the same site apparently evolving in nature and in SELEX. I would recommend omitting the section on the genetic code and replacing it with a discussion on reproducibility of SELEX, in which area several authors have done interesting work.

Response: *I agree that the issues with the genetic code are not so relevant to the particular problem of convergent versus divergent protein evolution. That section was included largely as a response to William Martin, reviewer of Koonin & Wolf's comment, where he stated that my original analysis was guilty of "ignoring the strength of the universality of the genetic code and the commonality of central intermediary metabolism among cells as evidence". The only point I wished to make was that, while the near-universality of the genetic code is strong evidence for UCA, it is currently infeasible to address with a formal model selection analysis — hence a rationale for using our well-tested and highly developed models of protein evolution instead. The convergent SELEX RNAs are certainly interesting and could in principle be analyzed in this framework, but they are less directly relevant to the question of UCA because they have not traditionally been used as an argument for UCA, whereas the universal genetic code has*.

The paper is generally well-written and, with the caveats above, in my opinion suitable for publication after revision. It might benefit from shortening, as some of the points are overly belabored in the current presentation. A more concise discussion focusing on the main ideas, i.e. the importance of model selection, the assumptions of the Koonin & Wolf model, and the consequences of these assumptions in distinguishing simulated and real data would likely increase readership substantially relative to the present version.

### Reviewer 2: Robert Beiko

(The review by Robert Beiko includes two consecutive rounds of comments, here interleaved. The second exchange is indented.)

In any event, the paper is certainly worthy of publication — I have some continuing problems with some details that I outline below, but this is a great contribution to an ongoing discussion.

This paper is a detailed response to Koonin and Wolf (ref [2]) in which a compositional model was used to generate phylogeny-free sequences to test the model selection framework of ref [1]. The paper consists of (1) A justification of the need for a formal test of common ancestry, (2) A detailed description of several critical limitations of null hypothesis-based tests of statistical significance, and (3) a detailed refutation of the work of Koonin and Wolf.

I think part (1) is fine; I have not followed the genetic code debate closely, but I agree that quantitative hypothesis tests based on evidence apart from the genetic code are worthwhile.

Part (2) addresses some well-covered areas of statistical theory, although the "Wolf vs. Koonin" etc. example is less illuminating than the rest of the section. I think e-values get short shrift, though: if one considers two different e-values from two different comparisons, I think one would be justified in taking the larger of the two e-values to constitute greater evidence for a lack of process generating similarity between the two sequences (convergence, common ancestry, etc.).

Response: *This is an exceedingly common misinterpretation of frequentist null-hypothesis significance tests. As explained in the text, by the very logic of null-hypothesis tests, a large E-value (or P-value) is evidence for nothing. This cautionary point is routinely reiterated in introductory statistical texts, e.g. "Although a 'significant' departure provides some degree of evidence against a null hypothesis, it is important to realize that a 'nonsignificant' departure does not provide positive evidence in favour of that hypothesis. The situation is rather that we have failed to find strong evidence against the null hypothesis." *[123]. *Similarly, "… a large significance level indicates that, with respect to the particular test used, the data provide no evidence that this hypothesis is false. This should not be interpreted as evidence in support of the hypothesis, but merely as a lack of evidence against it. " *[124]

Here is one way of understanding why it is fallacious to make conclusions from insignificant results. If the null hypothesis is true, then we expect to find probable results from the null distribution (i.e., we expect a large "insignificant" E-value, e.g. E > 0.1). But using probable results under the null to conclude that the null is true is committing the fallacy of affirming the consequent: if A then B, we observe B, therefore A — an invalid syllogism. In the case of sequence similarity tests, observing low similarity (e.g., a small S statistic in BLAST search) is expected under the null distribution of random alignments. But we also expect low similarity if the sequences are homologous and have diverged for a large amount of time.

Given the lack of well-developed alternative models to homology, it seems reasonable to me to consider a small BLAST e-value as constituting stronger evidence for homology than a larger one. I agree that this is not as strong as explicit model comparisons, but it does nonetheless highlight an important problem with the entire procedure as reported in the 2010 Nature paper and here.

Before I get to part (3), I have some major concerns about the approach, in particular how the tests are formulated and how the results are interpreted as positive evidence of common ancestry.

First and foremost, any likelihood or Bayesian model-contrasting framework is only as good as the models you feed to it.

Response: *This is true, but from a Bayesian point of view, this is not really a problem. Rather, it is a fundamental and inherent property of the scientific method itself, common to all methods of inference in general, and Bayesian methods handle it in the only proper way. We never know the true model, and in fact we can be certain that even our best models are wrong. To quote Box: "all models are wrong, but some models are useful" *[125]. *The purpose of a Bayesian analysis (and of science in general, in my opinion) is this: given a set of models hypothesized to explain our data, which model in this set should we believe is best at explaining the data? The most we can ever do is to say that one model is more probable than another (and quantify how much more probable)*.

The original 2010 paper contains the sentence "Statistically significant sequence similarity can arise from factors other than common ancestry, such as convergent evolution due to selection, structural constraints on sequence identity, mutation bias, chance, or artefact manufacture". But none of these alternatives is actually tested alongside the hypothesis of common ancestry!

Response: *I did indeed test some of those alternatives. In my original 2010 Nature paper I explicitly tested models of independent ancestry and chance convergence of the observed sequence similarity (as well as the nested, hierarchical patterns in the real protein data). Furthermore, my independent origin models are good approximations of independent origin models that include convergence due to several of those other factors (see below)*.

The independent origin tests use amino acid substitution models that were generated under the assumption of homology, and apply them to sequences that are completely homologous or very nearly so (see next paragraph). We seem to all be in agreement that these sequences are homologous (indeed the author says so in his response to Yonezawa and Hasegawa [116,120]: "neither selection nor physical constraints alone can plausibly generate the high levels of sequence similarity (55% average sequence identity) observed in the universal protein data set that I used."

Response: *My quoted claim is not equivalent to conceding that the sequences are homologous. There are other explanations, besides homology, for high sequence similarity. My statement was not an assumption but a conclusion based on my model selection results, taking into account realistic biophysical and biochemical considerations*.

This statement taken in isolation is fine, but is at odds with the claim that a meaningful, quantitative hypothesis test has been formulated and carried out, since no attempt has been made to develop plausible alternative models (for instance, models with independent origins + convergence) and let them fight it out with competing alternatives.

Response: *First, I did formulate and carry out a test with alternative models — *Figures 1b and 2b* from the original paper *[1]*are graphical representations of some of these alternative, independent ancestry models. The independent ancestry models that I considered could in principle produce the observed protein data, but the model selection tests show that the probability of chance convergence from independent origins is extremely low*.

*Second, my response to Yonezawa and Hasegawa *[120]*provides an explanation of why my independent ancestry models are reasonable approximations to models explicitly incorporating convergence due to physical constraints and selection for function. Any independent origin model incorporating selection and/or physical constraints must be based on the known biophysical and biochemical properties of proteins. The most pertinent characteristic of proteins is their extreme functional redundancy. To a good first approximation, primary sequence determines structure, and in turn structure determines function. Yet many sequences give the same structure, and many structures provide the same function *[120,126-128]. *All our biophysical and biochemical data indicate that the sequence space consistent with any particular function is so large as to be nearly indistinguishable from random background residue frequencies *[120,126-131]. *For selection to converge on similar sequences, sequence space must be tightly coupled to function space, so that independent selection pathways for the same function would be likely to result in similar sequences. In reality, however, there is an incredibly large sequence space associated with any particular function — so large that knowledge of the function alone tells us essentially nothing about the possible sequences that could specify that function*.

*In accord with these biophysical facts, each independent ancestry in my models has a prior on the residue frequencies given by their background frequencies (these can be provided a priori by the residue substitution matrices, but in my analyses they were actually inferred from the protein sequence data). Hence my independent ancestry models approximately account for (1) constraints on protein sequences imposed by function and (2) the prior probability of selection for function converging on similar sequences. For the same reasons, the independent ancestry models can also be considered as representing design hypotheses in which a "designer" intended to produce proteins with similar functions and/or structures, with an ignorance prior on the sequence space fulfilling those criteria (reflecting the fact that we know nothing about the intentions of the designer). For proteins with highly dissimilar sequences, it would be important to carefully and explicitly account for the very weak constraints on sequence imposed by function. But the proteins in my universal data set have relatively high sequence similarities. Hence the extremely large model selection scores strongly suggest that these approximations are reasonable, since subtle differences in background frequencies will have a negligible effect on the model selection scores reported in my Nature letter*.

Beiko response:

The "universal" proteins do indeed have high sequence similarities, but at the same time the ribosomal proteins you examined would seem to be an extreme case in comparison with the enzymes that were examined in e.g., [126]. The highly interacting nature of ribosomal proteins, along with their tendency to co-localize in large operons, would potentially make the probability of convergence quite a bit higher than for your typical garden-variety "active sites + a bunch of glue" enzyme. I agree that a proper model of independent origin of (at least some) ribosomal proteins + convergence would not be expected to tip the scales in terms of model evaluation, but the degree of model preference could easily shrink by quite a bit.

Response: *This is a good point — physical constraints, such as necessary physical interactions between ribosomal proteins and the ribosome, increase the probability of convergence (i.e., increasing constraints decrease the size of the relevant sequence and structure space consistent with a given function). For proteins with very weak similarities, these constraints may be important to model. In any case, my results are robust to omission of the ribosomal proteins*.

I mention "completely homologous" because there is a serious problem contained within at least some of the supplementary examples of the 2010 paper. Consider for instance the three sequences in Section 4.3, where E+M have significant sequence similarity, as do M+P, but not E+P. But looking at the BLAST alignments clearly shows these sequences for what they are: M is a multidomain protein that is partially homologous with E and partially homologous with P in a way that does not overlap with E. But the alignment columns are then shuffled, such that M+P are identical in columns 1,2,5,8,9, and so on, while E+M are identical at 3,4,6,7, etc. Were these sites properly clustered, inspection of the BLAST local alignments would immediately give a clear indication of what is going on. But since the columns are shuffled (and I know of no process that would give rise to this pattern of intercalated similarities), BLAST is railroaded into showing both local alignments as covering the entire protein length. This seems like an irrelevant example when the highly conserved proteins used in the main paper will show no such tendencies. I would like to know how the example in 4.2 was generated as well.

Response: *None of this matters for the intended point — these sequences were not intended to mimic proteins in my dataset, they were made to illustrate a methodological point. The point of the example in Section 4.3 is simply that the model selection tests prefer independent ancestry for certain highly similar sets of sequences (sequences with extremely statistically significant similarity, E ⋘ 10^-100^). Therefore the methodology considers factors other than mere sequence similarity in assessing independent vs common ancestry, contra Koonin and Wolf*.

The second problem is that the entire notion of common ancestry is ill-defined, especially in light of lateral genetic transfer. I think it is unlikely in the extreme that the components of the universal cellular machinery examined here are anything but homologous, but it seems to me that establishing common ancestry of each of these components does not guarantee that there was a single ancestral organism (or population of organisms, for any reasonable definition of 'population') in which all copies of the machinery were ancestral to the ones we would see today, which would seem to be necessary to make the leap from "common ancestry of a bunch of proteins" to "a common ancestor of life". This matter has been raised by Peter Gogarten, Olga Zhaxybayeva, Ford Doolittle, and Joel Velasco (pers. commun.), among others. What exactly is being formally tested here?

Response: *Universal common ancestry is defined here as "the proposition that all extant life is genetically related" *[1]. *The fundamental question I addressed was not whether the universally conserved genes in the Brown dataset are similar — they unquestionably are. Rather, I tested whether these sequences — and by necessity the organisms that carry them in their genomes — are genetically related by common ancestry. If you carry genes in your genome that are homologous to genes carried in the genomes of all other living organisms, then logically you are genetically related to all other living organisms. It is not simply that we have "common ancestry of a bunch of proteins". Rather, we have "common ancestry of a bunch of fundamental proteins that are each found in all known living organisms"*.

*Lateral genetic transfer is an orthogonal question. All life could be genetically related, via only vertical genetic inheritance from ancestor to descendant. Alternatively, all life could be genetically related, with rampant lateral gene transfer. There are many intermediate possibilities*.

The issue of the nature of the "universal common ancestor" is also largely independent of the status of universal common ancestry. For instance, Ford Doolittle speaks of common ancestry without a common ancestor. It will be useful to quote him at length:

*We (some of us) do doubt that there ever was a single universal common ancestor (a last universal common ancestor or LUCA), if by that is meant a single cell whose genome harboured predecessors of all the genes to be found in all the genomes of all cells alive today. But this does not mean that life lacks 'universal common ancestry' — no more than the fact that mitochondrial DNA and Y-chromosome phylogenies do not trace back to a single conjugal couple named Eve and Adam whose loins bore all the genes we humans share today means that members of Homo sapiens lack common ancestry. That 'common ancestry' does not entail a 'common ancestor' is perhaps a subtle point … *[15]

Similarly, I wrote in the original 2010 Nature letter:

*UCA does not demand that the last universal common ancestor was a single organism, in accord with the traditional evolutionary view that common ancestors of species are groups, not individuals. Rather, the last universal common ancestor may have comprised a population of organisms with different genotypes that lived in different places at different times*.

*The fact of the matter is that organisms existed in the past that contained ancestral copies of these fundamental proteins, and that all known extant life has inherited descendant versions of these proteins from these ancestral organisms (at least that is the scenario that my model selection tests strongly support). Whether you want to call that ancestral group of organisms "a species", "a population", "an ancestor", "the LUCA", or something else is primarily an issue of semantics. My universal common ancestry models are also consistent with a single individual organism/cell that contained all the ancestral versions of these proteins, though that is extremely unlikely given basic pop-gen considerations (and even less likely with LGT)*.

*Because all extant living organisms carry descendants of these fundamental proteins, at some point the ancestral group of organisms necessarily had the ability to exchange fundamental genetic material. This is one argument for calling this ancestral population a "species", regardless of whether it was exchanging genetic information via sex or LGT or whatever — e.g., that would be a strict application of the Biological Species Concept. Similarly, Doolittle and others have argued that "the proper way to model prokaryotic evolution over 4 Gyr is as a single, albeit highly structured, recombining population, not an asexual clade." *[15].

The rest of this review I dedicate to the Results and Discussion, part (3) of the paper. 3a, 3b and 3c refer to the three claims that are to be refuted (top of p. 15), 3a is further split into four parts.

3a-I, pp. 15-17: Of course phylogeny of a group of sequences models the hierarchical structure of a set of sequences in a way that simple pairwise BLAST cannot. But sequence similarity is evidently a major contributor to the optimal tree in terms of its branch lengths and topology, and based on the examples presented in the SI of the original Nature paper, it seems that one could use a graph-based approach to building putatively homologous sequence sets from cliques or almost-cliques of BLAST results. To me, it seems that extending the analysis of sequence similarity to sets (n > 2) of sequences may be the critical factor in the model test, rather than the following step of phylogenetic inference and modelling. Can a counterexample be constructed where most or all sequences in a set show statistically significant similarity to one another, yet the likelihood-based tests support independent ancestry over common ancestry?

Response: *Yes, and I presented exactly this type of counter-example in Section 4.3 of the Supplementary Information of my original paper. I have also constructed many other examples where three or four sequences all have highly significant sequence similarity to each other (rather than just two of three pairwise comparisons as in Section 4.3). In response I now present an additional four-sequence example in the text*.

3a-II, p. 17: Similarly to above, I don't think it's fair to say that the three proteins in the example in 4.3 of the Nature paper show "significant similarity" when only 66% of the sequences show such similarity.

Response: *Homology is transitive — if A and B are homologous, and B and C are homologous, then A is homologous to C. If significant sequence similarity is in fact a reliable marker for homology, then those three proteins should be homologous even though only two of three pairwise comparisons show significant sequence similarity. Analogous examples are commonly found in real proteins. Regardless, the new four-sequence example should mollify this concern*.

Beiko response:

Homology is clearly not transitive in the case of multidomain proteins. For example, if protein P1 has domains X+Y and protein P2 has domains Y+Z, it is not correct to say that a third protein P3 containing only domain Z is homologous to P1 because P2 happens to contain elements of both P1 and P3.

And this is clearly what is happening in the three- and four-protein examples: careful inspection of the proteins in Appendix 2, Additional File 1 shows that each column either supports a perfect partitioning of the sequence sets into BE+MP, or BM+EP, or BP+ME. So this example is equivalent to taking six distinct, non-homologous domains, and building sequences E = D1D3D5, M = D1D4D6, B = D2D3D6, P = D2D4D5 with odd/even pairs forced to sit on top of one another in an alignment. Shuffling the columns to generate the Appendix 2, Additional File 1 sequences allows BLAST to construct local alignments that span non-homologous "gaps" between the fragments of homologous domains, thus making it seem like the sequences are similar to each other in non-exclusive ways.

My point here is that these examples have similarity patterns that are evolutionarily implausible,

Response: *This is exactly the point. The patterns of similarity in these artificial sequences are highly unlikely to have been generated by evolutionary, phylogenetic processes as described by the common ancestry models. These patterns of similarity, however, could easily result if the sequences have independent origins (e.g., from some sort of convergent process or by design — the latter independent origin hypothesis is the correct one for these sequences). Whatever the case, the sequences are highly similar, and yet nevertheless the model selection tests (correctly) choose independent ancestry over common ancestry*.

and the argument that model comparisons capture more than simple sequence similarity are not bolstered by these artificial scenarios. The manner in which these examples were constructed could be described as "partial common ancestry", and raise the question of what model should be preferred when sequences are forcibly aligned in such a way that part of the alignment is true homology and part is not. The preference for BE+MP in Table 2 is presumably a consequence of more columns supporting this pairing than any other.

The 4.2 example is more confusing to me as I do not see how the likelihood is generated for the independent origin model. Is it based solely on stationary amino acid frequencies, without any branch lengths estimated? It seems to me that an example with two sequences is not ideal to illustrate the distinction between the two types of approach: were a third sequence added that was similar to 1&2 such that all pairs of the triad were significantly similar, I presume that common ancestry would once again be supported.

Response: *I agree that the 4.2 example is less applicable, so I have removed reference to it in the text*.

3a-III and IV, pp. 18-19: I do not think K&W suggested that the universal proteins could have been generated by a process lacking in phylogenetic structure:

Response: *As I read it, K&W implied that their profile model was a suitable model for the universal proteins, at least as far as my model selection tests were concerned. Otherwise, what is the relevance of their profile model to the actual data analyzed in my paper?*

rather, the point of their paper was that the key result of CA > IA could be generated from a dataset in which CA was not the correct model.

Response: *As I argued in detail in the text, K&W have never shown that their artificial dataset was not generated by a CA model*.

It also seems odd to contrast a star tree model with the partial independent origins model, when the natural complement to a star tree would be an independent origin for each of the sequences considered. I expect under those circumstances that the star tree would be favoured over a true independent origins model, as is the case with the K&W data.

Response: *I also expect that a star-tree would be preferred over independent origins for each of the sequences, but I don't find that to be a very informative comparison. In contrast, the star-tree vs independent ancestry tells us something important. Star-trees model similarity, but not nested hierarchical patterns. The independent origin models do the opposite; they model hierarchical patterns well, but not similarity between the independent groups. The key observation is that star trees are worse than independent origin models for the real proteins (as gauged by the model selection scores). This strongly suggests, then, that hierarchical structure is more important than similarity for the real protein data*.

Beiko response:

What I was getting at here is that in the partial independent origins model, hierarchical structure and similarity are still conflated at every level except that of the deepest interdomain relationships. And I would expect these relationships to have the smallest overall impact on the question of hierarchy vs. similarity, since they would be the least stressed out about having to coexist in a star tree, as opposed to much more similar sequences that desperately want to share an internal branch somewhere, anywhere. I don't think the distinction here is as clean as it is made out to be.

Response: *I agree the distinction is not completely clean. Nevertheless, a hypothesis that can describe some hierarchical structure, but absolutely no similarity between two groups ("partial" independent ancestry), does much better than a hypothesis that cannot model hierarchical structure, yet models similarity between the groups well (star tree). The independent ancestry hypothesis cannot account for any similarity between the two groups, whereas the star tree can, and yet the star tree, a common ancestry model, does much worse. Therefore, something in the independent ancestry hypothesis, something necessarily other than mere similarity, is responsible for the independent ancestry hypothesis beating the star tree. The only factor that could be responsible is hierarchical structure, and this is the case even though only the deepest interdomain relationships are really affected*.

Concerning the profile model, it's not surprising that the model under which the data were generated turns out to perform best. Clearly the K&W simulation was not intended to mimic the structure of the universal protein data set, but the fact remains that data sampled under residue probability distributions, with no true phylogenetic structure, generated a data set which led to an incorrect preference for the model presented in the original paper.

Response: *As I explained in the text, if the data are generated by a common ancestry model (as K&W's profile model is), and the tests choose common ancestry, then they chose correctly. And more importantly, when the true generating model is included in the set of models being tested, the model selection tests choose the true generating model*.

Also, please clarify why the amino acid dataset has a data-to-parameter ratio of 0.63.

Response: *For the profile model, each column in the alignment has 19 free parameters (the 20 residue frequencies that must be estimated, which sum to one). In the real, universal protein data set, there are only 12 sequences, so each column has only 12 observations from which to estimate the 19 parameters, giving a data-to-parameter ratio of 12/19 = 0.63*.

3b: Under "K&W's justification is faulty", two definitions of "independent" are conflated. What I take from the K&W quotation is that the patterns of similarity at each site are independent from one another, since there is no underlying tree. Conversely, the common assumption of "independently evolving sites" does not require that sites have uncorrelated histories or patterns of similarity, rather that changes at one site are not conditional on changes at other sites.

Response: *Admittedly the K&W quotation is somewhat vague as to just what they mean by "independent". However, it does not matter for my point if your interpretation is correct. Just because the patterns of similarity at each site are independent from one another does not mean that there is "no signal of common ancestry" or "no evolutionary signal", as K&W claim. As I mentioned in the text, a star tree produces patterns of similarity at each site that are independent from one another — and a star tree is a bona fide phylogeny, being an evolutionary representation of common ancestry*.

K&W themselves never claimed that their sequences were artificially convergent, only that they are highly similar.

Response: *Three counter-examples from their comment: "these alignments contain no signal of common ancestry (in more general terms, no evolutionary signal) whatsoever … ". "Alignments of statistically similar but phylogenetically unrelated sequences successfully mimic the purported effect of common origin." "The computational experiment on the estimation the likelihoods of alignments of similar but evolutionarily unrelated sequences." Sequences that are similar yet evolutionarily (or phylogenetically) unrelated are by definition convergent at the level of sequence *[132].

While these sequences were indeed sampled from homologous alignment columns, they certainly would possess what I would consider to be "conflicting phylogenetic signal". Indeed there seems to be some confusion about whether that term is meant to refer to reticulate evolutionary patterns within a single set of homologous genes, or the partial homology problem I point out above.

Response: *By "phylogenetic signal", I mean non-random, nested hierarchical patterns of similarities in the sequences. It is possible for a single set of sequences to have two or more very different, incompatible nested patterns of similarities, which is what I mean by "conflicting phylogenetic signal". This can occur in concatenated alignments of homologous proteins when there is reticulate evolution (since each protein is treated as one single long sequence). But it can also occur within a single protein (for instance reticulate evolution of domains in a multi-domain protein), or within a single protein domain. For my purposes the cause of conflicting phylogenetic signal is irrelevant — the point is that conflicting phylogenetic signal is highly unlikely to result from a common ancestry model (in which a local region of sequence is modelled with a phylogenetic tree). Regardless, any apparent conflicting phylogenetic signal found in K&W's artificial data is necessarily only due to random chance*.

The comparison of the sampling protocol of K&W to the models of Bruno is very interesting, although the fact that the generating columns had hierarchical structure to begin with makes me wonder whether the analogy is accurate or not. In any case, does this mean that we cannot in fact distinguish homologous proteins from nonhomologous proteins that exist in a reduced but shared sequence space due to structural constraints?

Response: *In some cases, I think the answer is yes. However, imagine we have a reduced sequence space and yet the actual sequences show strong nested, hierarchical patterns. In that case the common ancestry models should explain the data better than independent ancestry models (better than models that account for the reduced sequence space yet cannot account for hierarchical patterns), and so we should be able to distinguish the two*.

3c: You should probably add the assumption that the sequences used to generate the substitution models (JTT, WAG, etc.) were themselves homologous, and emphasize the assumption that the Markovian process is stationary, reversible, and homogeneous.

Response: *I've modified the text regarding the Markovian assumptions. But the substitution models deserve some comment, and I have added a section of clarification in the text. First, for the formal purposes of the model selection tests, we don't actually know that the substitution models (like WAG) were based on homologous sequences, and this assumption is unnecessary. The only thing we really know is that the sequences used in constructing WAG, etc. were similar. From a model-selection perspective, the origin of the substitution matrices is irrelevant — the likelihood function doesn't know where the matrices came from, and the likelihood is the final judge. All that matters is that some substitution matrices give higher likelihoods than others*.

Beiko response:

But the WAG matrix was built on the underlying assumption that a phylogenetic tree related the sequences whose changes contributed to the entries in the matrix. If WAG was in fact forcing similar but non-homologous sequences to obey a phylogenetic framework, wouldn't that have some impact on your contrasting of models that have varying components of phylogenetic relatedness?

Response: *The WAG matrix is, ultimately, nothing more than a proposed set of substitution rates. Regardless of where those rates came from, they may work well in the evolutionary models or they may not (relative to other rate matrices). More importantly, perhaps, is the fact that we can dispense with an assumed rate matrix altogether and use Mr Bayes to infer a general time reversible amino acid rate matrix from the universal protein data itself. I have done such with the universal proteins; it doesn't change the results. And the matrix inferred from the actual data is qualitatively quite close to WAG*.

These assumptions seem particularly relevant if we are testing common ancestry of sequences that may have initially emerged in a period of reduced amino acid diversity and other drastic differences in evolutionary processes. I'm not an expert on early amino acid alphabets, but it seems that contrasting CA vs. IA at a time when there were 10 or fewer amino acids in use would drastically change the degree to which different models are preferred, especially if a meaningful model for convergence could be developed.

Response: *These considerations are worth keeping in mind. But since all of these proteins (from widely divergent taxa) have the same 20 amino acids and the same genetic code, the most parsimonious explanation is that the genetic code (and amino acid alphabet) was set in its modern form well before the divergence of this set of proteins (however, Syvanen offers an alternative view that arginine and tryptophan were added rather late *[30]).

### Reviewer 3: Michael Gilchrist

This paper is a response to a critique of the author's previous high profile paper in Nature [1]. Theobald (2010) claimed to have quantitatively evaluate the hypothesis that there is a single, universal common ancestor for all of extant life. The author did this by comparing the ability of alternative models to explain the sequence similarity observed in a set of 'universal' proteins. The critique by Koonin and Wolf [2] argued that Theobald's "… demonstration of the universal common ancestry is a trivial consequence of significant sequence similarity between the analyzed proteins." Koonin and Wolf go further to assert that the ability to demonstrate universal common ancestry (UCA) "… is unlikely to be feasible in principle." Two of the reviewer's of this paper were also quite critical of the Theobald paper, arguing its result is either based on circular reasoning or 'trivial' in nature.

In the current paper Theobald addresses these criticisms by arguing that they are either incorrect, in the case of whether or not his model will be preferred by model selection even if it is incorrect, or misleading, in the fact that Koonin and Wolf's data is simulated in a manner consistent with common ancestry, but in a non-nested manner (i.e. a star tree). In the end I find Koonin and Wolf's 'demonstration' of Theobald's approach to be poorly thought out, failing in fact to demonstrate what they claim, and Theobald's response to be thoroughly convincing. (I guess this should not be unexpected if you can choose your reviewers). Further, I see no philosophical reason why the UCA hypothesis cannot be tested and find myself convinced that this is the best explanation of the data given the set of models currently being proposed.

In terms of criticisms, I find the author's definitions of P-values and E-values to be so similar that the reader is left a bit confused about how they differ.

Response: *P-values and E-values are indeed quite similar, and in my experience most people find the subtle difference between P-values and E-values to be confusing and arcane. For the purpose of this paper the similarity is the important thing — the difference does not matter, as there is a one-to-one correspondence between P-values and E-values, they have the same philosophical statistical justification, and they are used identically in null-hypothesis significance tests*.

I think the author could improve the paper by revisiting the section where he states "Now imagine that you replace these six sequences …" Stating that the likelihood will change if you change the data seems trivial and, given how models are evaluated in terms of relative differences in likelihood, confusing as to the main point.

Response: *It may seem trivial to those familiar with the likelihoods of probabilistic phylogenetic models. But the point is important: it is possible to change the sequence data so that the similarity is unchanged. If the likelihood changes but the similarity does not, then the phylogenetic models, and hence the model selection scores, necessarily gauge something besides mere sequence similarity, contra K&W*.

In addition instead of saying "… the likelihood of a phylogenetic tree is also a function of the particular patterns of characters … " (particular patterns is a bit vague) it might be better to say "… the likelihood of a phylogenetic tree is also a function of the nested nature of the similarities in the data… "

Response: *The sentence mentioned has been changed as suggested*.

Overall, I think that it is interesting to see the clash of scientific philosophies on display between the 'old guard' of Koonin and Wolf (and reviewers) whose scientific interpretation and training appears to be centred on null hypotheses and the 'new guard' of Theobald whose work is focused on model selection.

## Competing interests

The author declares that he has no competing interests.

## Authors' contributions

DLT carried out all work and wrote the manuscript.

## Supplementary Material

Additional file 1 **Appendix 1 and 2**. Click here for file
