# Supplementary material for: On universal common ancestry, sequence similarity, and phylogenetic structure: the sins of P-values and the virtues of Bayesian evidence
Source: Biol Direct. 2011 Nov 24;6:60. doi: 10.1186/1745-6150-6-60 (PMC3314578; doi:10.1186/1745-6150-6-60)
Supplement: Additional file 1 — Appendix 1 and 2. [file 1745-6150-6-60-S1.PDF]

Appendix

Marginal likelihood of the profile model

The likelihood at each site j (or column in the alignment) of N sites is described by a categorical distribution:

$$p(\mathbf{X}_j|\mathbf{q}_j, \mathbf{H}) = \prod_{i=1}^{K} {q_{ji}}^{x_{ji}} \tag{3}$$

where $\mathbf{X}_j$ is the observed sample of amino acid residues at site j, $K = 20$ for amino acids, and $\mathbf{q}_j$ is a vector of probability parameters for the amino acid frequencies ($\mathbf{q}_j$ has elements $q_i, i = 1 \ldots K$), and

$$\sum_{i=1}^{K} q_{ji} = 1. \tag{4}$$

Because each site is independent, the total likelihood is the product of the N individual site likelihoods:

$$p(\mathbf{X}|\mathbf{q}, \mathbf{H}) = \prod_{j=1}^{N} p(\mathbf{X}_j|\mathbf{q}_j, \mathbf{H}) \tag{5}$$

I assume a conjugate Dirichlet prior for the vector of probability parameters, as the Dirichlet is the natural conjugate prior for the categorical distribution [1]. The prior distribution for each site is described by:

$$p(\mathbf{q}_j|\alpha_j, \mathbf{H}) = \frac{\Gamma\left(\sum_i^K \alpha_{ji}\right)}{\prod_i^K \Gamma\left(\alpha_{ji}\right)} \prod_{i=1}^{K} {q_{ji}}^{\alpha_{ji}-1} \tag{6}$$

where α_j is the vector of hyperparameters for the Dirichlet. Recall that a marginal likelihood is found by integrating the product of the likelihood and the prior for the parameter:

$$p(\mathbf{X}|\mathbf{H}) = \int p(\mathbf{X}|\theta, \mathbf{H})\, p(\theta|\mathbf{H})\ d\theta \tag{7}$$

In this case the marginal likelihood of the categorical model has an analytical solution. The marginal likelihood for each site j is given by (omitting the j index for clarity):

$$p(\mathbf{X}_j|\mathbf{H}) = \int_0^1 \frac{\Gamma\left(\sum_i^K \alpha_i\right)}{\prod_i^K \Gamma\left(\alpha_i\right)} \prod_{i=1}^{K} {q_i}^{x_i+\alpha_i-1}\ d\mathbf{q} \tag{8}$$

$$= \frac{\Gamma\left(\sum_i^K \alpha_i\right)}{\prod_i^K \Gamma\left(\alpha_i\right)} \int_0^1 \prod_{i=1}^{K} {q_i}^{x_i+\alpha_i-1}\ d\mathbf{q} \tag{9}$$

$$= \frac{\Gamma\left(\sum_i^K \alpha_i\right) \prod_i^K \Gamma\left(x_i+\alpha_i\right)}{\Gamma\left(\sum_i^K [x_i+\alpha_i]\right) \prod_i^K \Gamma\left(\alpha_i\right)} \tag{10}$$

The Perks prior is a common uninformative “ignorance” prior used with categorical and multinomial distributions, a diffuse “unit information prior” equivalent to the prior information imparted by a single observation [2]. For the Perks prior, $\alpha_i = \frac{1}{K}$, so:

$$\mathrm{p}(\mathbf{X}_j|\mathbf{H}) = \frac{\prod_i^K \Gamma\left(x_i + \frac{1}{K}\right)}{\Gamma\left(1 + \sum_i^K x_i\right) \Gamma\left(\frac{1}{K}\right)^K} \tag{11}$$

Due to site independence, the total marginal likelihood is the product of the N individual site marginal likelihoods:

$$\mathrm{p}(\mathbf{X}|\mathbf{H}) = \prod_{j=1}^{N} \mathrm{p}(\mathbf{X}_j|\mathbf{H}) \tag{12}$$

Significantly similar sequences that favor independent ancestry

```
>esco1
EAPDWEPPAAAQNHQHPAWAAPHNQWWDHQRNNRRAARRDDDEQRHWDQDAWNREDQDWD
NWPWRRADEADPDQRQEPWHWWAPPPARRWRDHDNHNDADHNRHHRDQPPQRWWADRAHQ
AAPPNWWQRAWDRRHDNHRNNEHPRWWPARHQANADHAAPEQNQRWNANAAWAAWPWWAW
RRAWPPEHNRWWERPRQHHAWWWDRPRAWEPRRNWARAERANNWRWAWDNQAREQQWRRR
NAEDDHEQHRWHWDRNADRNARNQWRPNDAQRNWWQQWWEAWPPNWQWQRQHRRWRHEQA
AAWDRERPDHAPDRARQDAWDRWNRHRRQENAPAPNWQAHHWAAAAARAWPWWERWWAWR
AHPRWRRWWPHAEEPDREAPRDARHWRERDDWAAANDRPEHDRDRRWQPERWQWHPARAA
RHQRRWANWRNHRQAANANRDWARRHPRRQEQWNERRPEAAQPRDRQAANWRWRWNPRNA
HHARWQWWAARANERPARWHDRRWWWHRNQHNWRAQAHWRWAWHANRHWAWRQRDPQWRW
>mytu1
FRPDPFPPRRRINHIHPCPRCPHNIDPDHIFNNFFCCFFDDDFIIHPDIDCPNIFDIDPD
NPPDIIRDFCDPDIFIFPPHDPCPPPCFIPFDHDNHNDCDHNIHHFDIPPIIDPCDFCHI
RRPPNPDIICDDIFHDNHINNFHPFPPPRFHICNRDHCRPFINIFPNCNRCDRRPPDDCD
FFCDPPFHNIDDFIPFIHHCDPPDFPIRPFPFFNDRIRFICNNDIDRDDNIRIFIIDFIF
NRFDDHFIHIDHPDFNRDFNCINIDFPNDRIINPDIIDPFRPPPNDIDIFIHFIPFHFIC
RCDDIFIPDHRPDFRFIDCPDIPNIHFIIFNRPRPNDICHHDRCCRRFRDPPDFIPDCPF
CHPFPFIPPPHRFFPDIFRPIDCIHDFFIDDDRCRNDFPFHDIDIFPIPFIDIDHPCIRR
FHIIFPCNPFNHFIRRNRNFDPCFIHPFFIFIDNFFIPFCCIPFDIICCNPFDIDNPINC
HHCIPIPPRCFCNFFPCFDHDFFDDPHFNIHNDICIRHPFDCPHRNFHPRDFIFDPIPFD
>basu1
FAYYWFYYAAAIGGIGYAWAAYGGIWWYGIFGGFFAAFFYYYFIIGWYIYAWGIFYIYWY
GWYWIIAYFAYYYIFIFYWGWWAYYYAFIWFYGYGGGYAYGGIGGFYIYYIIWWAYFAGI
AAYYGWWIIAWYIFGYGGIGGFGYFWWYAFGIAGAYGAAYFIGIFWGAGAAWAAWYWWAW
FFAWYYFGGIWWFIYFIGGAWWWYFYIAWFYFFGWAIAFIAGGWIWAWYGIAIFIIWFIF
GAFYYGFIGIWGWYFGAYFGAIGIWFYGYAIIGWWIIWWFAWYYGWIWIFIGFIWFGFIA
AAWYIFIYYGAYYFAFIYAWYIWGIGFIIFGAYAYGWIAGGWAAAAAFAWYWWFIWWAWF
AGYFWFIWWYGAFFYYIFAYIYAIGWFFIYYWAAAGYFYFGYIYIFWIYFIWIWGYAIAA
FGIIFWAGWFGGFIAAGAGFYWAFIGYFFIFIWGFFIYFAAIYFYIIAAGWFWIWGYIGA
GGAIWIWWAAFAGFFYAFWGYFFWWWGFGIGGWIAIAGWFWAWGAGFGWAWFIFYYIWFW
>pogi1
ERYYPEYYRRRQGGQGYCPRCYGGQDPYGQRGGRRCCRRYYYEQRGPYQYCPGREYQYPY
GPYDRRRYECYYYQRQEYPGDPCYYYCRRPRYGYGGGYCYGGRGGRYQYYQRDPCYRCGQ
RRYYGPDQRCDYRRGYGGRGGEGYRPPYRRGQCGRYGCRYEQGQRPGCGRCDRRPYDDCD
RRCDYYEGGRDDERYRQGGCDPPYRYRRPEYRRGDRRRERCGGDRDRDYGQRREQQDRRR
GREYYGEQGRDGPYRGRYRGCRGQDRYGYRQRGPDQQDPERPYYGDQDQRQGRRPRGEQC
RCDYRERYYGRYYRRRQYCPYRPGRGRRQEGRYRYGDQCGGDRCCRRRRDYPDERPDCPR
CGYRPRRPPYGREEYYRERYRYCRGDRERYYDRCRGYRYEGYRYRRPQYERDQDGYCRRR
RGQRRPCGPRGGRQRRGRGRYPCRRGYRRQEQDGERRYECCQYRYRQCCGPRDRDGYRGC
GGCRPQPPRCRCGERYCRDGYRRDDPGRGQGGDRCQRGPRDCPGRGRGPRDRQRYYQPRD
```

References

1. Bernardo JM, Smith AFM: Bayesian theory. Chichester, Eng.: Wiley 1994.
2. Perks W: Some observations on inverse probability including a new indifference rule. Journal of the Institute of Actuaries 1947, 73:285-334.
